# Chronic Active Epstein–Barr Virus Infection: Is It Immunodeficiency, Malignancy, or Both?

**DOI:** 10.3390/cancers12113202

**Published:** 2020-10-30

**Authors:** Shigeyoshi Fujiwara, Hiroyuki Nakamura

**Affiliations:** 1 Division of Hematology and Rheumatology, Department of Medicine, Nihon University School of Medicine, Tokyo 173-8610, Japan; 2 Department of Allergy and Clinical Immunology, National Research Institute for Child Health and Development, Tokyo 157-8535, Japan; nakamura-hry@ncchd.go.jp

**Keywords:** Epstein–Barr virus, EBV, chronic active EBV infection, hydroa vacciniforme, severe mosquito bite allergy, EBV-positive T/NK-cell lymphoproliferative disease, immunodeficiency, lymphoma

## Abstract

**Simple Summary:**

Chronic active Epstein–Barr virus (EBV) infection (CAEBV) is a rare syndrome of unknown etiology characterized by prolonged infectious mononucleosis-like symptoms and proliferation of EBV-infected T and/or natural killer cells. CAEBV has been primarily reported in East Asia and Latin America, suggesting a genetic predisposition in its pathogenesis. The clinical course of CAEBV is heterogeneous ranging from an indolent and occasionally self-limiting disease to an aggressive and fatal condition, but its prognosis is generally poor. This heterogeneous clinical picture does not suggest a simple etiology for the syndrome. Clinicopathological investigations of CAEBV suggest that it has aspects of both malignant neoplasm and immunodeficiency. This article summarizes the latest findings on CAEBV and discusses critical unsolved questions regarding its pathogenesis and disease concept.

**Abstract:**

Chronic active Epstein–Barr virus (EBV) infection (CAEBV) is a rare syndrome characterized by prolonged infectious mononucleosis-like symptoms and elevated peripheral blood EBV DNA load in apparently immunocompetent persons. CAEBV has been primarily reported in East Asia and Latin America, suggesting a genetic predisposition in its pathogenesis. In most cases of CAEBV, EBV induces proliferation of its unusual host cells, T or natural killer (NK) cells. The clinical course of CAEBV is heterogeneous; some patients show an indolent course, remaining in a stable condition for years, whereas others show an aggressive course with a fatal outcome due to hemophagocytic lymphohistiocytosis, multiple organ failure, or progression to leukemia/lymphoma. The pathogenesis of CAEBV is unclear and clinicopathological investigations suggest that it has aspects of both malignant neoplasm and immunodeficiency. Recent genetic analyses of both viral and host genomes in CAEBV patients have led to discoveries that are improving our understanding of the nature of this syndrome. This article summarizes the latest findings on CAEBV and discusses critical unsolved questions regarding its pathogenesis and disease concept.

## 1. Introduction

Epstein–Barr virus (EBV) is a ubiquitous herpes virus carried by more than 90% of the adult population worldwide [1]. Primary EBV infection occurs most often in childhood and is usually asymptomatic, but when it occurs in adolescents or young adults, infectious mononucleosis (IM), a self-limiting lymphoproliferative disease, develops at a frequency of 25–74% [2]. EBV-positive B-cell lymphoproliferation and exaggerated T-cell responses to the virus are thought to play central roles in the pathogenesis of IM. Regardless of being accompanied by IM or not, acute primary EBV infection results in life-long asymptomatic persistent infection with intermittent virus release into the saliva [1]. In contrast, EBV was the first identified human tumor virus and is etiologically associated with a number of malignancies and lymphoproliferative diseases, including Burkitt lymphoma, Hodgkin lymphoma, nasopharyngeal carcinoma, gastric carcinoma, and lymphoproliferative disorders (LPDs) of immunocompromised hosts [1].

The main targets of EBV infection are B lymphocytes and epithelial cells. Infection of the former usually leads to latent infection, whereas that of the latter predominantly results in viral replication. EBV establishes life-long persistent infection in memory B cells [1]. EBV has a unique biologic activity that growth-transforms B lymphocytes into lymphoblastoid cells that have the potential to proliferate autonomously in vivo [1]. Virally-encoded proteins involved in the process of B-cell growth transformation are generally considered to play central roles in the pathogenesis of neoplastic diseases associated with the virus [3]. In contrast to the high efficiency with which EBV growth-transforms B cells and establishes lymphoblastoid cell lines, infection of EBV to T cells and natural killer (NK) cells is inefficient. Although there have been a few reports describing experimental EBV infection of thymocytes [4] and some T-cell lines [5], it has not been possible to growth-transform T/NK cells with EBV in vitro.

## 2. Chronic Active Epstein–Barr Virus Infection and Related EBV-Positive T/NK-Cell LPDs and Leukemia/Lymphomas

### 2.1. Chronic Active EBV Infection

IM is clinically characterized by various manifestations including fever, pharyngitis, lymphadenopathy, and hepatitis. Although these manifestations of IM usually subside within a few weeks to months [2], in rare cases, they may persist or recur repeatedly for more than a few months to years after onset [6,7,8,9,10,11]. These cases present heterogeneous conditions in terms of clinical manifestation and prognosis, and have historically led to a broad spectrum of new medical terms, including chronic infectious mononucleosis [6], chronic mononucleosis syndrome [10], and chronic symptomatic EBV infection [11]. A fraction of these patients follow a more severe and even fatal clinical course, with high anti-EBV antibody titers and/or EBV DNA load, hepatosplenomegaly, multi-organ failure, and complications resulting from leukemia/lymphoma. Patients have occasionally exhibited symptoms suggesting minor defects in immune response to the virus [7,10,12,13,14]. This more severe category largely corresponds with chronic active EBV infection (CAEBV) (more precisely systemic CAEBV (see below)). The prognosis of CAEBV is generally poor and hematopoietic stem cell transplantation is the only curative therapy [15,16].

In the most recent WHO classification of tumors of hematopoietic and lymphoid tissues, CAEBV is defined as a “systemic EBV-positive polyclonal, oligoclonal, or (often) monoclonal lymphoproliferative disorder characterized by fever, persistent hepatitis, hepatosplenomegaly, and lymphadenopathy, which shows varying degrees of clinical severity depending on the host immune response and the EBV viral load” [17]. The diagnostic criteria of CAEBV include IM-like symptoms persisting for >3 months, increased EBV DNA (>10^2.5^ copies/μg DNA) in peripheral blood, histological evidence of organ disease, and demonstration of EBV RNA or viral proteins in affected tissues in patients without known immunodeficiency, malignancy, or autoimmune disorders [17,18,19,20]. CAEBV cases have been primarily reported in East Asia (Japan, Korea, China, and Taiwan) and Latin America (indigenous people in Central/South America and Mexico), and in these areas, proliferation of EBV-infected T or NK cells has generally been observed (CAEBV of T/NK-cell type) [21,22,23,24]. In contrast, in Western countries including the USA and Europe, the incidence of CAEBV is much lower and proliferation of EBV-infected B cells has predominantly been observed (CAEBV of B-cell type) [7,19,25]. However, there have been rare cases of patients with B-cell type CAEBV in East Asia [26]. This uneven geographical distribution suggests that a genetic predisposition is involved in the pathogenesis of CAEBV, although no definitive information has been obtained so far. In the WHO classification mentioned above, CAEBV of the T/NK-cell type is divided into systemic and cutaneous forms, with the latter category subdivided into hydroa vacciniforme (HV)-like LPD and severe mosquito bite allergy (SMBA) [17]. The clinical manifestation of CAEBV, including both T/NK-cell type and B-cell type, is heterogeneous. Some patients follow a mild and indolent clinical course and remain stable for years or may recover spontaneously (e.g., the classic form of HV-like LPD), whereas others follow an aggressive course with a rapid fatal outcome due to serious complications such as hemophagocytic lymphohistiocytosis (HLH), multi-organ failure, and progression to leukemia/lymphomas [26,27,28]. Although CAEBV was originally considered a pediatric disease, recent studies have identified increasing numbers of adult-onset cases that display a poorer prognosis as compared with pediatric cases [29,30].

### 2.2. EBV-Positive T/NK-Cell LPDs and Leukemia/Lymphomas Related to CAEBV

Since the unexpected detection of EBV in the T and NK cells of CAEBV in the late 1980s [21,22,23], a number of related LPDs and leukemia/lymphomas have been shown to exhibit EBV-positive T- or NK-cell lymphoproliferation, including hydroa vacciniforme (HV), severe mosquito bite allergy (SMBA), extranodal NK/T-cell lymphoma, nasal type (ENKTL), and aggressive NK-cell leukemia (ANKL). HV is a photoreactive cutaneous childhood disorder characterized by herpetiform vesiculopapules on sun-exposed areas [31]. Although HV is largely self-limiting and resolves spontaneously (classic HV), it occasionally develops into systemic illness similar to systemic CAEBV (systemic HV) [31]. In the recent WHO classification, a spectrum of disease ranging from classic to systemic HV was given the umbrella term “HV-like LPD” [17]. EBV-positive T cells (or occasionally NK cells) are observed in the skin lesions and peripheral blood of both classic HV and systemic HV patients [31]. SMBA is a mosquito bite-triggered skin reaction characterized by erythema, edema, bullae, and deep ulceration accompanied by systemic manifestations including fever, lymphadenopathy, and hepatosplenomegaly [32]. Both HV-like LPD and SMBA can present as cutaneous manifestations of systemic CAEBV.

ENKTL is an NK or T-cell lymphoma of extranodal localization characterized by cytotoxic phenotype, vascular destruction, and local necrosis [33,34]. ANKL is a rare leukemia of mature NK-cell lineage characterized by an aggressive and fulminant clinical course [35,36]. Both ENKTL and ANKL can develop in the clinical course of CAEBV. Clinicopathological features of these EBV-positive leukemias/lymphomas, together with other rare categories of EBV-positive T/NK-cell lymphomas, including systemic EBV-positive T-cell lymphoma of childhood and EBV-positive nodal peripheral T-cell lymphoma, have been thoroughly reviewed in recent articles [34,36]. All of the EBV-positive T/NK-cell LPDs and leukemias/lymphomas related to CAEBV have similar geographical distributions restricted to East Asia and Latin America.

## 3. CAEBV as a Malignant Neoplasm

Clonal proliferation of EBV-infected cells is observed in CAEBV, suggesting their malignant nature, although morphological atypia of these cells is not observed in typical cases. Monoclonality of EBV-infected cells does not necessarily indicate a poorer prognosis for the patient in CAEBV [28]. On the other hand, overt malignancies such as ENKTL and ANKL may develop in the clinical course of CAEBV. In this context, as Ohshima et al. suggested, CAEBV may be understood as “a particularly indolent form of T/NK-cell malignancy with a tendency to evolve into a more aggressive neoplasm” [28]. The age distribution of patients with the extranasal form of ENKTL shows a bimodal curve, with one major peak at middle age and another minor one at young-adult age. Takahashi et al. suggested that the three conditions, (i) EBV-positive NK/T-cell lymphoma of extranasal localization with the onset at young-adult age, (ii) ANKL, and (iii) NK-cell-type CAEBV with overt lymphoma (type A3 of [28]), comprise a continuous spectrum of diseases that are difficult to distinguish on a clinicopathological basis [37]. Here, the history of CAEBV episodes is the only distinction between NK-cell CAEBV of type A3 and the other two conditions [37]. Although the relationship between ENKTL of middle-age onset and CAEBV is unclear, a 56-year-old patient with ENKTL that developed after a prodromal period consistent with CAEBV has been reported [38], suggesting that a small fraction of ENKTL with middle-age onset may also develop on the basis of CAEBV. A fraction (18/113, 16%) of patients with ANKL exhibited a subacute clinical course with a better prognosis. These patients had a prolonged prodromal phase with IM-like manifestations for more than 90 days, revealing similarities between this subacute type of ANKL and CAEBV [39]. It appears difficult to clearly demarcate a border between CAEBV and malignancies such as ENKTL and ANKL.

A recent comprehensive genomic study of CAEBV revealed that a substantial fraction of CAEBV patients carried somatic mutations in the so-called “driver” genes that are known to play critical oncogenic roles in various malignant neoplasms [40]. Okuno et al. reported that EBV-positive cells carried mutations in driver genes including *DDX3X* (14 of 80 cases (18%)), *KMT2D* (4 of 80 cases (5.0%)), and *BCOR*/*BCORL1* (3 of 80 cases (3.8%)) [40]. These mutations were shown to occur in tumors such as Burkitt lymphoma and ENKTL. CAEBV patients with these driver mutations exhibited poorer survival rates than those without [40]. These results indicate that a fraction of CAEBV cases should be considered malignant neoplasm cases. A question of interest is whether the frequency of these driver mutations is higher in adult patients with CAEBV than in pediatric cases, which might explain a worse prognosis in the former. This study [40] also presented the finding that EBV genomes residing in the EBV-infected cells of CAEBV frequently carry intragenic deletions. These deletions frequently occur in the *Bam*HI rightward transcript (BART) region encoding the BART microRNA clusters and also occur in several essential genes required for viral replication. Since BART microRNAs are known to target the EBV immediate-early genes *BZLF1* and *BRLF1*, deletions in the BART region are expected to enhance initiation of the viral lytic cycle [41]. On the other hand, deletions in essential replicative genes preclude completion of the viral lytic cycle and consequent cell death. It is presumed that these deletions as a whole increase the chance of an abortive lytic cycle. Recent studies have indicated that higher levels of TNF-α, CCL5, and IL-10 are produced from EBV-transformed lymphoblastoid cells with higher levels of lytic activation [42]. These cytokines are thought to inhibit functions of cytotoxic lymphocytes and recruit immunosuppressive myeloid cells, and thereby enhance oncogenesis [43,44,45]. 

## 4. CAEBV as Immunodeficiency 

### 4.1. Analysis of Immune Functions in CAEBV

Cellular immune responses by both cytotoxic T lymphocytes (CTLs) and NK cells play critical roles in the immune control of EBV infection and defects in cellular immunity predispose infected hosts to EBV-positive LPDs and leukemia/lymphomas [3,46]. Although the diagnostic criteria for CAEBV include the absence of known immunodeficiency, detailed analyses of patient immune function have revealed minor defects in cellular immunity against EBV-infected cells [47,48,49]. Tsuge et al. examined two patients with NK-cell-type CAEBV and found that while their CTL activities against both EBV-infected B cells and NK cells were markedly decreased, the frequency of CTL precursors specific to EBV-infected B cells remained normal. In contrast, CTL precursors specific to EBV-infected NK cells were undetectable, suggesting some mechanism for escaping immunological surveillance on EBV-infected NK cells [48]. A quantitative study using human leukocyte antigen HLA class I tetramers also revealed a drastically reduced frequency of EBV-specific CD8+ T cells in CAEBV; CD8+ T cells specific to LMP2 were not detectable in any of the eight patients examined [49]. The frequency of CD8+ T cells specific to the HCMV protein pp65 was also lower, suggesting that the defective T-cell response in CAEBV is not specific to EBV [49]. One study revealed defective activity in EBV-specific CTLs not only in patients with CAEBV but also in their parents, suggesting a hereditary nature of the defect [47]. Defects have also been revealed in the NK-cell activity of patients with CAEBV [50,51]. In one study, NK-cell cytotoxicity was deficient in two CAEBV patients in the same family and in an EBV-seronegative healthy individual within the family, again suggesting a genetic element to this immune defect [51].

An obvious question is why this supposed immunodeficiency leads to proliferation of EBV-infected T/NK cells and not B cells in CAEBV of the T/NK-cell type. One possible explanation is the difference in EBV latent gene expression in T/NK cells and B cells. EBV-infected T/NK cells in CAEBV have been shown to typically exhibit the latency II pattern of viral gene expression, in which EBV proteins such as EBNA1, LMP1, and LMP2 are expressed [52,53,54]. Here, immunodominant proteins such as EBNA2 and EBNA3s, which are frequently recognized by EBV-specific CTLs, are not expressed. In contrast, in EBV-immortalized B cells, all EBV latent proteins including EBNA2 and EBNA3s are expressed. It is therefore conceivable that EBV-infected T/NK cells may be more prone to escaping from immunosurveillance in the minor immunodeficiency supposed in CAEBV. In this context, the selective deficiency of LMP2-specific CTLs observed in patients with CAEBV could have a significant negative impact on the immunosurveillance of EBV-infected T/NK cells [49]. In a rare B-cell type CAEBV case in Japan, a unique restricted EBV gene expression in which only EBNA1 was expressed as a viral protein was documented [53]. Absence of EBNA2 and EBNA3s in these cells may have rendered them more prone to escaping from CTLs [53].

### 4.2. Clinical Manifestations Consistent with B-Cell-Type CAEBV in Patients with Primary Immunodeficiency

Reflecting the critical importance of cellular immunity in the control of EBV infection, patients with various primary immunodeficiencies, especially those exhibiting defects in cellular immunity, have a high risk of developing EBV-positive LPDs and leukemia/lymphomas, and some of these patients show clinical manifestations consistent with CAEBV [55,56,57]. However, in contrast to typical cases of CAEBV, most of these patients develop opportunistic infections not only with EBV but also with other pathogens, and primarily display lymphoproliferation of EBV-positive B cells. Thus, clinical features of B-cell-type CAEBV have been observed in patients with mutations of various genes including *PRF1* [58], *UNC13D* [59], *STXBP2* [59,60], *PIK3CD* [61], *MAGT1* [62], *ITK* [63], *GATA2* [64], *CD70/CD27* [65,66,67], and *CTPS1* [68] (reviewed in [56,57]).

Cohen et al. investigated 19 patients with CAEBV who were diagnosed in the United States from 1982 to 2010 [19]. These patients included 11 cases of B-cell type, 3 cases of T-cell type, 1 case of NK-cell-type CAEBV, and 4 cases of CAEBV in which the type of EBV-infected cell was undetermined. Major clinical manifestations including fever, lymphadenopathy, splenomegaly, and hepatitis, as well as only transient success with chemotherapy, were similar to those found in CAEBV patients in East Asia and Latin America. However, these USA cases were characterized by progressive loss of B cells and hypogammaglobulinemia, especially in B-cell-type CAEBV. They also indicated that cellular immunotherapy using autologous EBV-specific CTLs, which is effective in the treatment of post-transplant LPD (PTLD), was not effective against CAEBV, suggesting impaired T-cell function in these patients. Other immunological analyses of B-cell-type CAEBV have also revealed signs of immunodeficiency, which are most likely of a hereditary nature [7,25]. Frequent association with obvious immunodeficiency and apparently unrestricted geographical distribution suggest that B-cell-type CAEBV is etiologically distinct from the T/NK-cell type.

### 4.3. Primary Immunodeficiency Accompanied by EBV-Positive T/NK-Cell LPDs

Although the vast majority of EBV-positive LPDs found in individuals infected with HIV or given immunosuppressive drugs are of B-cell origin, EBV-positive T/NK-cell LPD has also been documented with low incidence in these immunocompromised conditions [69,70]. Similarly, while most cases of EBV-positive LPDs observed in patients with primary immunodeficiency (PID) were of B-cell origin as stated above, several recent studies have identified patients with PID presenting EBV-positive T/NK-cell lymphoproliferation, and some of them have exhibited clinical features consistent with CAEBV, as shown in Table 1.

*GATA2* encodes a transcription factor critically involved in hematopoiesis and immune function and one patient with HV-like LPD exhibited haploinsufficiency of *GATA2* due to unialleleic expression [64]. In addition, EBV-positive peripheral T-cell non-Hodgkin lymphoma developed in a patient who carried two heterozygous mutations (G28fs and H26P) of *GATA2* [71]. Since clinical manifestations are highly variable among individuals carrying the same *GATA2* mutation, influence from mutation or variation in other “modifier” genes is suspected [71].

The *IL2RG* gene encodes the common γ chain of various cytokine receptors and its loss-of-function (LOF) mutation causes X-linked severe combined immunodeficiency (X-SCID). A hypomorphic mutation of *IL2RG* (c.C982T, p.R328*) was identified in a male patient with EBV-positive γδT-cell LPD. He had apparent signs of immunodeficiency (low T- and NK-cell numbers, low T/NK-cell proliferative response, and deficiency in STAT3/5/6 phosphorylation following stimulation with cytokines) and therefore was not diagnosed with CAEBV [74].

Hypomorphic mutations of the *SH2D1A* gene (c.G7T, p.A3S) and the *XIAP* gene (c.1045_1047delGAG, p.E349del) were identified in patients with T/NK-cell-type CAEBV from Japan. Since the deleterious effects of these mutations on the functions of encoded proteins seem minimal, the role of the mutations in CAEBV may depend on the presence of additional genetic factors [75]. A patient with common variable immunodeficiency due to a homozygous mutation of the *FANCA* gene (c.190_191insT (p.E65RfsX5)) was reported to develop CAEBV of the NK-cell type [73]. A patient with a homozygous *CD27* mutation (c.G158A, p.C53Y) developed EBV-positive T-cell LPD that progressed into T-cell lymphoma [72].

Recently, Rodriguez et al. reported that a patient with T-cell type CAEBV, conceived during a consanguineous relationship, had two homozygous LOF mutations: one in the *TNFRSF9* gene (c.170delG, p.G57fsX91) and the other in the *PIK3CD* gene (c.2462G > A, p.R821H) [76]. *TNFRSF9* encodes the cell surface protein CD137 and *PIK3CD* encodes the catalytic subunit of the phosphoinositide 3-kinase, p110δ. From immunological analyses of the patient, the authors surmised that the *PIK3CD* mutation enhanced the proliferation of EBV-infected T cells and the mutation of *TNFRSF9* impaired clearance of EBV-infected T cells by cytotoxic T cells. Interestingly, an asymptomatic sister of the patient also exhibited an elevated EBV-infected T-cell level comparable to that in the patient. Because she had the same homozygous LOF mutation in *TNFRSF9* but intact *PIK3CD*, this finding suggests that the mutation of *TNFRSF9* by itself can lead to the increase in EBV-infected T cells in the peripheral blood. This study also illuminated the possibility that multifactorial genetic inheritance may underlie the pathogenesis of at least some CAEBV cases. 

Although only a limited number of PID patients with EBV-positive T/NK-cell LPDs have been analyzed immunologically, further research in this area may reveal critical immune defects that preferentially permit proliferation of EBV-infected T and NK cells. The aforementioned examples of EBV-positive LPDs observed in patients with mutations/variations in *GATA2* [71], *SH2D1A* [75], *XIAP* [75], *TNFRSF9/PIK3CD* [76], and *STXBP2/PRF1* [60] suggest that concomitant mutations/variations in multiple immune-related genes may be involved in the pathogenesis of EBV-positive LPDs. Even a common variation of immune-related genes (e.g., the A91V variation of *PRF1* that is present in up to 5% of Caucasians) may contribute to the pathogenesis of B-cell-type CAEBV, if accompanied by mutations in certain other genes [60].

## 5. Unsolved Questions in CAEBV

### 5.1. Mechanisms of EBV Infection to T and NK Cells

Some fundamental questions need to be addressed to understand the pathogenesis of CAEBV and related EBV-positive T/NK-cell LPD/lymphomas. Although the mechanisms of EBV entry into B cells and epithelial cells are well understood, those of EBV entry into T/NK cells are unclear. EBV entry into B cells starts with interactions between the EBV envelope protein gp350 and CD21 and between the envelope proteins gH/gL/gp42 and an HLA class II molecule [1]. These interactions lead to internalization of the virus particle via membrane fusion mediated by gH/gL and gB [1]. The entry of the virus into epithelial cells involves interactions between the viral envelope protein BMRF2 and cellular integrins (α1, α3, α5, and αv) and between gH/gL and integrins (αvβ6 and αvβ8), resulting in membrane fusion mediated by gH/gL and gB [1]. T cells and NK cells are not thought to express significant levels of the EBV receptor CD21. Although these cells express some integrins, no evidence has yet been obtained that suggests EBV infection to these cells occurs via integrins. Since EBV-infected T/NK cells in CAEBV patients consistently express cytotoxic molecules, including T-cell restricted intracellular antigen 1 (TIA1), granzyme B, and perforin; infection via immunologic synapses formed between EBV-infected B cells and cytotoxic T or NK cells during target cell lysis has been proposed as a possible mechanism [28,77].

A fraction of patients with CAEBV harbor EBV-infected cells of multiple lineages, e.g., T cells and NK cells. Southern blot analysis of viral DNA in these patients identified a clonal band containing the same number of terminal repeats, suggesting that EBV infected immature or progenitor lymphoid cells that have a potential to differentiate into both T and NK cells [78]. A recent genomic analysis of somatic mutation in CAEBV revealed clonal evolution of EBV-infected cells in a fraction of patients and strongly suggested that lymphoid progenitors that have the potential to differentiate into various lymphocyte lineages are infected with EBV and then differentiate into B, T, or NK cells [40]. There is evidence suggesting that immature T cells or lymphoid progenitor cells can be infected with EBV [79,80]. It is therefore likely that, in some CAEBV patients, EBV infects immature T cells or lymphoid progenitor cells, although experimental confirmation is necessary. EBV-infected T or NK cells have been found not only in CAEBV or related EBV-positive T/NK-cell LPD/lymphomas but also in a substantial fraction of patients with IM, indicating that EBV infection of T/NK cells does not necessarily result in pathogenic T/NK-cell proliferation [81,82]. The development of T/NK-cell LPDs including CAEBV may require EBV infection to unusual host cells such as lymphoid progenitors. 

EBV can be classified into two genotypes, EBV-1 and EBV-2, based on sequence differences in the genes encoding EBNA2 and EBNA3s [1]. EBV-1 is prevalent worldwide and transforms B cells efficiently, whereas EBV-2 is prevalent in a limited geographical range and less efficiently transforms B cells [1]. A recent study detected the expression of CD21 on mature human peripheral T cells using a particular monoclonal antibody (HB5) and indicated that EBV-2 can infect T cells via interactions between the CD21 molecule and the viral glycoprotein gp350 [83,84]. However, EBV-2 is not prevalent in East Asia and all CAEBV-derived T/NK-cell lines have been shown to harbor EBV-1 [85]. Experimental infection of EBV-1 to T or NK cells is inefficient, if not impossible [83].

### 5.2. Restricted Geographic Distribution

There are three possible explanations for the restricted distribution of CAEBV in East Asia and Latin America. The first is a specific EBV strain that is predisposed to inducing T/NK-cell proliferation and has a geographic distribution similar to that of CAEBV. Although early studies suggested the presence of such EBV strains [86,87], they were not confirmed in subsequent studies [88]. The second is an environmental factor that has a similar geographical distribution to that of CAEBV. Although this hypothesis is formally possible, no plausible candidates for such a factor have been suggested, except for the possible involvement of pesticides in the pathogenesis of ENKTL [89]. The third possibility, which has been explored the most extensively, is genetic predisposition with uneven geographic distribution.

As mentioned above, minor immunodeficiency is presumed to be involved in the pathogenesis of CAEBV. If this immunodeficiency is determined by genetic predispositions that have uneven geographical distribution similar to CAEBV, this may provide an explanation for the restricted distribution of the disease. Recently, Ito et al. reported that the risk of CAEBV is associated positively with HLA-A26 and negatively with B52; curiously, both alleles are frequently found in East Asia and Latin America [90]. Variants of *SH2D1A* (p.Ala3Ser) (rs148554414) and *XIAP* (p.Glu349del) (rs199683465) that have been found in Japanese patients with CAEBV or EBV-HLH have a higher frequency in East Asia [75,91] (https://gnomad.broadinstitute.org/), although these variants have not been found in many of the CAEBV patients analyzed by next generation sequencing. Because de novo mutations are supposed to occur randomly and are not likely to explain the uneven geographic distribution of CAEBV, it may be necessary to investigate variant alleles that have biased geographic distributions similar to CAEBV. 

A geographical distribution restricted to East Asia and Latin America is observed not only in CAEBV but also in most other EBV-positive T/NK-cell LPD/lymphomas, suggesting a common genetic predisposition to these diseases. This supposed common genetic factor is most likely associated with a subtle impairment in immune control in EBV-positive T/NK-cell proliferation, although other possibilities including non-immune mechanisms cannot be excluded.

### 5.3. The Origins of Clinical Heterogeneity

The extreme heterogeneity of clinical manifestations and prognosis of CAEBV is a perplexing characteristic. Heterogeneity may be partially explained by differences in the stage of the disease at diagnosis because CAEBV is a progressive disease with complications developing in its clinical course. If diagnosed at an early stage, the disease may appear more indolent and benign, whereas if diagnosed at later stages, possibly with accumulated driver mutations in EBV-infected cells, it may appear more malignant and aggressive. Patients diagnosed after a long interval following disease onset are expected to have worse prognoses. A question of interest is whether the duration between disease onset and diagnosis tends to be longer in adult-onset CAEBV cases than in pediatric-onset cases.

Clinical heterogeneity may reflect the heterogeneity of etiology. A substantial number of patients have been examined by whole exome or genome analysis to find a genetic hallmark of CAEBV, but so far, no genetic mutation or variation common to the majority of patients has been identified [40,92]. This strongly suggests that CAEBV is a genetically heterogeneous disease, involving different genes or different set of genes in different patients. This heterogeneity in inheritance is expected to result in different clinical manifestations.

Clinical manifestations and prognosis are different in T-cell and NK-cell types of CAEBV; patients with T-cell-type CAEBV tend to have higher incidences of fever and anemia, and higher EBV antibody titers, whereas those with NK-cell-type CAEBV have higher incidences of large granular lymphocytosis, severe mosquito bite allergy, and an increased serum IgE level [54]. Somatic mutations of driver genes in EBV-infected lymphocytes are also heterogeneous among patients and may explain some of the clinical heterogeneity of CAEBV [40]. Furthermore, intragenic deletion observed in EBV genomes residing in CAEBV cells is also heterogeneous and may contribute to the clinical heterogeneity [40].

## 6. Concluding Remarks

**Figure 1:**
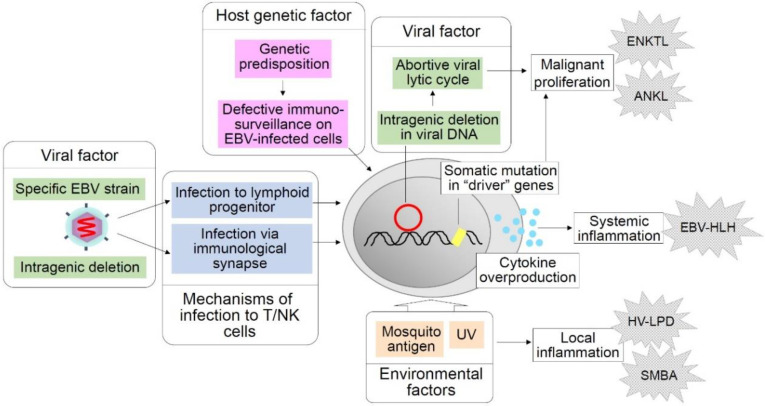
Hypothetical factors and events in chronic active Epstein–Barr virus (CAEBV) pathogenesis. Shown above are factors and events supposed to play critical roles in the pathogenesis of CAEBV and related EBV-positive T/NK-cell LPDs that may develop in the clinical course of CAEBV. Host genetic factors are supposed to impair immunosurveillance on EBV-infected cells. A possible viral strain predisposed to T/NK-cell lymphoproliferation is considered a viral factor. Intragenic deletions of the EBV genome that facilitate an abortive lytic cycle are presumed to enhance oncogenesis. Mosquito antigens and UV light are environmental factors that are thought to induce local inflammation such as hydroa vacciniforme (HV)-like LPD and severe mosquito bite allergy (SMBA). Infection to lymphoid progenitor cells and infection via immunological synapses are hypothetical mechanisms of EBV infection to T/NK cells.

CAEBV is a complex and heterogeneous syndrome with features of both immunodeficiency and malignant neoplasm. The exact mechanisms of CAEBV pathogenesis are still unclear and a number of questions remain to be answered. A hypothetical representation of CAEBV pathogenesis based on the discussions in this article is illustrated in Figure 1. As discussed above, a number of factors likely contribute to the clinical heterogeneity of the disease, including the nature of genetic predisposition and the stage of the disease at diagnosis. In addition, other factors such as the type and extent of somatic mutation of host driver genes, the type of intragenic deletion of the EBV genome, and the lineage of EBV-infected cells may provide additional variability to its clinical manifestations. Further research on these factors will more clearly define CAEBV and enable categorization of patients into further homogeneous subgroups. Targeted therapies for each subgroup of CAEBV patients are expected to result in more favorable outcomes. 

## Figures and Tables

**Table 1:** Mutations/variations in immune-related genes detected in patients with Epstein–Barr virus (EBV)-positive T/NK-cell lymphoproliferative disorders (LPDs).

Affected Gene	Type of Mutation/Variation	EBV-Related Manifestation	EBV-Unrelated Manifestation	Reference
*GATA2*	Haploinsufficiency due to unialleleic expression	HV-like LPD HLH	*Enterococcus faecium* bacteremia, infections with *Mycobacterium avium* complex and histoplasma; neutropenia, lymphopenia, reduced numbers of B cells and NK cells, hypogammaglobulinemia	[64]
	Heterozygous mutations (c.G28fs and p.H26P)	EBV-positive non-Hodgkin T-cell lymphoma	Pancytopenia	[71]
*CD27*	Homozygous mutation (c.G158A, p.C53Y)	EBV+ T-cell LPD developing into lymphoma	Oral ulcer, uveitis, recurrent non-EBV infections	[72]
FANCA	Homozygous mutation (c.190_191insT, p.E65RfsX5)	NK-cell-type CAEBV	Common variable immunodeficiency (hypogammaglobulinemia, sinusitis)	[73]
*IL2RG*	Hemizygous hypomorphic point mutation (c.C982T, p.R328*)	EBV-positive γδT-cell LPD	Recurrent respiratory infection, *Yersinia enteritis*, infection with *Haemophilus influenzae* Low T-cell count, complement deficiency (C2, C9), reduced mitogen-induced proliferation, dysgammaglobulinemia	[74]
*SH2D1A*	Hemizygous hypomorphic mutation/variation (c.G7T, p.A3S)	NK-cell-type CAEBV (indolent), photosensitivity, SMBA	No apparent immunodeficiency or other infections	[75]
*XIAP*	Hypomorphic mutation/variation (c.1045_1047delGAG, p.E349del)	NK/B-cell type CAEBV and SMBA in a hemizygous boy	No apparent immunodeficiency or other infections	[75]
		EBV-HLH following primary infection in a hemizygous boy	No apparent immunodeficiency or other infections	
		NK/CD4+ T-cell type CAEBV and SMBA in a heterozygous woman	No apparent immunodeficiency or other infections	
*TNFRSF9/PIK3CD*	Homozygous LOF mutation in TNFRSF9 (c.170delG, p.G57fsX91); homozygous LOF mutation in PIK3CD (c.2462G > A, p. R821H)	T-cell type CAEBV, HV-like LPD, EBV-HLH	Recurrent respiratory and skin infections (panaritium and boils), no apparent signs of immunodeficiency	[76]
*TNFRSF9*	Homozygous LOF mutation in TNFRSF9 (c.170delG, p.G57fsX91)	EBV+ T-cell lymphoproliferation without symptoms in the healthy sister of the patient described in the row above	No apparent signs of immunodeficiency	[76]

HV: hydroa vacciniforme. HLH: hemophagocytic lymphohistiocytosis. NK: natural killer. CAEBV: chronic active EBV infection. SMBA: severe mosquito bite allergy. LOF: loss of function.
